# Feasibility and Acceptability of an Adolescent-Friendly Rap Video to Improve Health Literacy Among HIV-Positive Youth in Urban Peru

**DOI:** 10.1007/s10461-020-03098-4

**Published:** 2020-11-17

**Authors:** Carly A. Rodriguez, Alexander Winnett, Milagros Wong, Neha Krishnam, Nicole Ocasio Martínez, Lady J. Perez, Lenka Kolevic, Leonid Lecca, Molly F. Franke

**Affiliations:** 1 Department of Global Health and Social Medicine, Harvard Medical School, 641 Huntington Avenue, Boston, MA 02115 USA; 2 Socios En Salud Sucursal Peru, Lima, Peru; 3 School of Public Health, University of Washington, Seattle, WA USA; 4 Department of Biology, University of Massachusetts Boston, Boston, MA USA; 5 Infectious Disease, Instituto Nacional del Salud del Niño, Lima, Peru

**Keywords:** Peru, Adolescent health, HIV, Health literacy, Health education

## Abstract

Clinical outcomes among adolescents living with HIV (ALHIV) might be improved by interventions aimed at addressing limited health literacy. We developed a Spanish-language rap video on HIV concepts and examined its acceptability and feasibility as a learning tool among ALHIV in Lima, Peru. Twenty-eight ALHIV receiving care at an urban pediatric hospital and ten stakeholders engaged in the care of adolescents watched the video. Adolescents completed a pre- and post-video questionnaire. We conducted focus groups with ALHIV and in-depth interviews with stakeholders and analyzed transcripts to identify themes. ALHIV described concepts of CD4 cell count and viral load as they were portrayed. Participants reported the video was relatable, accessible, and provided hope that ALHIV could lead healthy lives and advocated for future videos to address topics such as transmission and sexual health. Questionnaires indicated some improvement in viral load knowledge. An HIV health literacy music video intervention was feasible to implement and accepted by ALHIV and their healthcare providers. Communicating HIV knowledge via music videos may be promising; further study is needed to optimize implementation.

## Resumen

Los resultados clínicos entre los adolescentes que viven con el VIH (AVVIH) podrían mejorarse mediante intervenciones dirigidas a abordar la limitada alfabetización sanitaria. Desarrollamos un video de rap en español sobre los conceptos del VIH y examinamos su aceptabilidad y viabilidad como herramienta de aprendizaje entre los AVVIH en Lima, Perú. Veintiocho AVVIH que reciben atención en un hospital pediátrico urbano y diez interesados involucrados en la atención de adolescentes vieron el video. Los adolescentes completaron un cuestionario previo y posterior al video. Realizamos grupos focales con AVVIH y entrevistas a profundidad a los interesados y analizamos las transcripciones para identificar los temas. Los AVVIH describieron conceptos de recuento de células CD4 y carga viral tal como se retrataron. Los participantes informaron que el video era identificable, accesible y brindaba la esperanza de que los AVVIH pudiera llevar una vida saludable y abogaron por videos futuros para abordar temas como la transmisión y la salud sexual. Los cuestionarios indicaron cierta mejora en el conocimiento de la carga viral. Una intervención de video musical para educación en salud sobre el VIH fue factible de implementar y fue aceptada por los AVVIH y sus proveedores de atención médica. La comunicación de conocimientos sobre el VIH a través de videos musicales puede ser prometedora; se necesitan más estudios para optimizar la implementación.

## Introduction

Declines in HIV virologic suppression and adherence to combination antiretroviral therapy (cART) are common during adolescence [1–3], a time during which HIV management ideally becomes more autonomous in preparation for the transition to adult care [4]. HIV-related health literacy is essential for living healthily with HIV during adulthood: higher health literacy correlates with better combined antiretroviral therapy (cART) adherence and outcomes [5–8]. Despite increasing calls for “adolescent-friendly” services [9, 10], there are few studies on strategies for improving health literacy among adolescents living with HIV/AIDS (ALHIV). Alternative forms of communication, such as music and social media [11–14], may be effective for disseminating health messages to adolescents.

Despite a disproportionate burden of new HIV infections in Latin America and the Caribbean among 15–24 year olds [15], the majority of rigorously-evaluated interventions for adolescents and youth are conducted in high-resource settings or sub Saharan Africa. Across five systematic reviews of interventions implemented over the period 2001–2018 to improve linkage, retention, and adherence to HIV care among adolescents and youth [9, 16–19], no interventions were identified among ALHIV in Latin America and only one intervention was implemented in the Caribbean [20]. Evidence on culturally-tailored interventions targeted at ALHIV in Latin America is urgently needed.

To inform adolescent-friendly interventions addressing health literacy, we developed a Spanish-language rap video and assessed its acceptability and feasibility as a learning tool among ALHIV in Lima, Peru.

## Methods

### Video

Our prior work among ALHIV in Lima identified that unsuppressed viral load, CD4 count decline, and nonadherence increase throughout adolescence [2, 21]. We also found cART misinformation was an important barrier to adherence [22]. Therefore, our objective for the five-minute video was to describe CD4 cell count, HIV viral load, and cART adherence, and their interrelation. The Spanish-language lyrics were drafted by a team of six bilingual young adults and reviewed by key stakeholders, including study investigators and local clinicians. The video was filmed in different locations throughout Lima, set to the background music of a popular rap song, and featured the same team of young adults that drafted the lyrics.

### Participants

In May and June 2018, we enrolled 28 ALHIV (10–18 years) receiving HIV care at a large urban pediatric hospital in Lima, Peru and ten health care providers who routinely cared for adolescents, including pediatricians, infectious disease specialists, nurses, peer counselors, and the director of a non-profit organization providing community-based HIV services. Nearly all ALHIV were perinatally-infected. No adolescents or stakeholders refused participation; however, one adolescent who consented was unavailable on the day of the focus groups. Adolescent participants and an accompanying guardian were provided with lunch on the day of the session and reimbursed for travel (approximately 8 USD); stakeholders were remunerated approximately 10 USD for interviews.

### Procedures

Adolescents and their caregivers were approached to participate during routinely scheduled visits at the pediatric hospital. Participants were asked to watch the rap video. Adolescents completed a pre- and post-video questionnaire and participated in a focus group to share their opinions about the video. Key stakeholders completed in-depth interviews to discuss their opinions on video acceptability and utility, implementation strategies, and topics for future music videos. We defined acceptability as how well the intervention was received by ALHIV and how the intervention met the needs of ALHIV [23]. We followed Bowen’s model to assess feasibility [24]. We sought to determine whether the intervention could be implemented and whether the intervention was appropriate for future effectiveness testing [24, 25].

#### Quantitative Pre- And Post-Video Questionnaire

The pre- and post-video questionnaire was designed to assess comprehension of the video’s key HIV messages, including CD4 cell count, viral load and cART adherence. While the pre- and post-video questionnaire can be used as a preliminary assessment of the intervention’s effectiveness of improving health literacy, the study was not powered to detect within-person differences in responses. We purposefully sampled questions from validated tools to align with key themes. These included questions from The Brief Estimate of Health Knowledge and Action—HIV version (BEHKA-HIV) and HIV Treatment Knowledge Scale, a validated tool for youth living with HIV [26, 27]. Additionally, we included original questions related to key themes. The questionnaire was developed in English, translated to Spanish by a fluent study team member (MFF), and reviewed for accuracy by a native Spanish-speaker (MW). All questionnaires were provided to adolescents in Spanish.

#### Qualitative Focus Groups and In-Depth Interviews

A trained qualitative researcher led four focus groups with adolescents and conducted in-depth individual interviews with key stakeholders, which were audio-recorded. Semi-structured guides were developed for focus groups and interviews separately. To assess acceptability, focus group guides questioned adolescents on what they liked and disliked about the video and the perceived importance and applicability of video content to adolescents’ lives. Feasibility of the video primarily focused on if and how adolescents would watch or share the video. In-depth interview guides with key stakeholders focused on assessing acceptability via questions on the relevance and value of video content for ALHIV. We probed stakeholders on the feasibility of the intervention with questions on how the video could be shared with ALHIV or implemented in the future.

Guides were designed to assess acceptability, effective communication of key messages, importance of content, potential uses, and themes for future videos [23, 25]. All focus groups and interviews were conducted in Spanish and lasted approximately 45 min.

### Data Analysis

#### Quantitative Pre- and Post-Video Questionnaires

We assessed HIV knowledge acquisition by categorizing participants’ responses as correct or incorrect in the pre- and post-video questionnaire administration, resulting in four categories: incorrect at both time points, correct at both time points, correct in the pre-period but incorrect in the post-period, and incorrect in the pre-period but correct in the post-period.

#### Qualitative Focus Groups and In-Depth Interviews

Audio recordings were transcribed verbatim, translated by a bilingual study team member, and analyzed. One researcher read the transcriptions and grouped the data into main themes based on focus group guides (i.e., acceptability, effective communication of key messages, importance of content, potential uptake and use, and themes for future videos) and subgroup themes. Another researcher performed a secondary review to confirm findings. Discrepancies between researchers were resolved by discussion.

### Ethical Approvals

Study materials were reviewed and approved by the ethics committee at the National Institute of Child Health (Instituto Nacional de Salud del Niño, INSN), Lima, Peru and the institutional review board of Harvard Medical School, Boston, USA. The study was performed in accordance with the ethical standards as laid down in the 1964 Declaration of Helsinki and its later amendments or comparable ethical standards.

## Results

### Demographics and Self-reported Adherence

Of the 28 adolescents enrolled, 27 (96%) completed both the pre- and post-video questionnaire. Most adolescents were female (63%) and the median age was 15 years (25^th^–75th percentile: 13–16) (Table 1). Over half (16/25, 64%) of participants reported they were “good” or “very good” at taking their medications as directed in the last 30 days; none reported they were “excellent” at taking their medications. Similarly, the majority (21/25, 84%) of participants reported they “always” or “almost always” took their medications as directed in the last 30 days.

**Table 1:** Participant baseline characteristics and pre-video questionnaire responses related to adherence (N = 27, unless otherwise noted)

	n (%)
**Demographics**	
Female	17 (63%)
Age group	
12–13 years	8 (30%)
14–15 years	10 (37%)
16–17 years	9 (33%)
**Adherence**	
In the last 30 days, number of days with at least one missed dose of cART^a^ (N = 25)	2 (0, 2)
In your opinion, in the last 30 days, how well do you think you took your medications, as directed by your doctor? (N = 25)	
Very poor	0
Poor	0
Neutral	9 (36%)
Good	11 (44%)
Very good	5 (20%)
Excellent	0
In the last 30 days, how often did you take your medications correctly? (N = 25)	
Never	0
Rarely	1 (4%)
Sometimes	3 (12%)
Almost always	11 (44%)
Always	10 (40%)
I don’t take my medications when they make me feel bad (N = 25)	
I agree	4 (16%)
I do not agree	18 (72%)
I am not sure	3 (12%)
I don’t take my medicines when I am too tired (N = 25)	
I agree	2 (8%)
I do not agree	19 (76%)
I am not sure	4 (16%)
I don’t take my medicines when I am feeling down or low (N = 25)	
I agree	1 (4%)
I do not agree	21 (84%)
I am not sure	3 (12%)
I don’t take my medicines because it tastes bad (N = 25)	
I agree	3 (12%)
I do not agree	19 (76%)
I am not sure	3 (12%)
I don’t take my medicines when I feel good (N = 24)	
I agree	2 (8%)
I do not agree	20 (83%)
I am not sure	2 (8%)
I will stay healthy if I choose not to take my medications (N = 24)	
I agree	2 (8%)
I do not agree	21 (88%)
I am not sure	1 (4%)
I know what will happen if I do not take my medications (N = 24)	
I agree	22 (92%)
I do not agree	1 (4%)
I am not sure	1 (4%)
I will stay healthy if I forget to take my medication (N = 24)	
I agree	2 (8%)
I do not agree	19 (79%)
I am not sure	3 (13%)

*cART* combined antiretroviral therapy

^a^Median, 25th percentile–75th percentile

## Qualitative Focus Groups and In-Depth Interviews

Of the 28 adolescents enrolled, 26 (93%) participated in the focus groups; ten stakeholders, including seven pediatricians or infectious disease doctors, one nurse, one peer counselor, and one director of a non-profit organization, participated in in-depth interviews.

### Acceptability

Adolescents and stakeholders described how three aspects of the video enhanced its acceptability: relatable content, an adolescent-friendly medium, and ease-of-access.

#### Relatable Content

Participants explained how the familiar backdrop of the video and the diversity of the people featured in the video fostered a perception of personal relevance. One health provider commented on how the recognizable backdrop was significant to adolescents:

I think that it approaches their reality because there are images of Peru, a place they recognize. In other words [the setting] is not a foreign place where [the adolescents] say, ‘well maybe there yes, but here, no.’ Or that in those places, yes you are going to live, but here you are not. It’s saying that here, yes, there is a solution to the problem, this problem.

Adolescents enthusiastically responded to seeing places they recognized. One adolescent commented: “*[I like that] they chose places from here, from our country… the north, it’s all really good”*, while another adolescent expressed her joy seeing one of her favorite places and mentioned “*I loved [the video] because it is at my favorite beach.”*

Others appreciated the diversity of the artists in the video. A health provider described how the diversity of people helped adolescents feel like they were more relatable: “*They have included a diverse group, diverse races, men and women together, everyone singing together. I think this is good because anyone can feel identified.”* Another health provider commented on the effectiveness of peers (i.e., adolescents and young people) as communicators of information because “*even though they are already familiar with the illness, and they know what we tell them every day at their appointments, it’s always necessary for them to be reminded of something in their own language.”*

#### An Adolescent-Friendly Medium

Adolescents and stakeholders identified the audio and visual components of the video as attractive to the target population. Adolescents expressed their preference to listen to music and watch videos and described how the music engaged them:

[I like the video format] because it teaches me and I am going to learn through listening and it is not as boring as a brochure or a talk.

[The video is] interesting because they have used new music … a genre called trap. And they seem to have copied that song, a song that mostly young people listen to.

Health providers described how music was relevant to adolescents and effective in capturing their attention:

…this type of video clip is what usually attracts attention. I mean, unfortunately, I do not know if this is true in all countries, but the custom of reading things is very much lost. That is one thing that for me is wrong, but, well, that's our reality. If you give me something to read and something to watch that has music, I prefer [the latter]. That is very clear.

It gives them another medium that corresponds to their age and music that they know. And this is important, right? Hearing it from a peer that speaks to them, sings to them. They probably think they are alone with this illness.

#### Ease-of-Access to Video

A key consideration related to acceptability was whether adolescents were able to access the video. Health providers expressed that a web-based video would be broadly accessible to adolescents:

Well, they have YouTube… they can look for it, if we tell them, ‘look at this link or that one.’ And most of the kids now use the internet, no? I think if we say to them ‘if you want, you can watch it at home or you can repeat it and watch at the internet cafe’, then that would also be a reinforcement so that they know and that they watch it.

They could look for the video in many ways [including] internet cafes. They go to the places where there is Wi-Fi, with tablets, computers, laptops. Ay, right now most of them have access to the internet, they have a link.

#### Effective Communication of Key Messages

Two key themes emerged in this area: (1) adolescents reported improved understanding of key concepts in HIV, as they were portrayed in the video, and (2) health providers highlighted aspects of the video facilitating this effective communication.

Both qualitative data and quantitative data suggest adolescents improved their understanding of the role the CD4 count, viral load, and cART adherence. When asked what he understood from the video, this adolescent responded by using a metaphor from the video:

…That we have to take care of our health and that our CD4 must increase and that our … viral loads will go down and our defenses will win and put the virus to sleep in the body.

Another common takeaway was the importance of cART adherence for viral load suppression and overall health. One adolescent explained that, for her, the importance of adherence was a key take-away:

We have to take the pills or the [antiretrovirals] so that everything goes well, and that is the truth. If we take the medication, we are not going to get sick and we will be able to control our viral load too. If we take the medication, the viral load is controlled and there won’t be any problems.

Adolescents juxtaposed their knowledge of CD4 count and viral load before and after watching the video. These two adolescents reported the video corrected misconceptions and offered new information:

Adolescent: *Before the video, I thought that CD4 was for example, the virus that was in our body and the viral load was something good.*

Study worker: *And now in the video, what are you understanding?*

Adolescent: *That the CD4 is our cells that protect our body.*

Study worker: *And the viral load?*

Adolescent*: Our virus that is acting.*

Adolescent: *It’s interesting.*

Study worker: *Interesting…did you learn something about the topic?*

Adolescent: *Yes, about CD4, what CD4 is.*

Study worker: *And what is it?*

Adolescent: *One’s defenses*

Study worker: …*And was there another idea that you captured from the video…?*

Adolescent: *That one has to conquer HIV.*

Study worker: *That one has to conquer HIV, and how are you going to conquer HIV?*

Adolescent: *Taking our medicines and paying attention to the doctor.*

#### Delivery of Key Messages

Health providers described aspects of the video facilitating effective messaging, including repetition, a succinct message, and a motivating tone:

…They repeat [the message] several times and that is good. The more you repeat the message the more you will internalize it. It is also not good to put many messages in the song, two or three main ideas is what I noted. They are about the CD4 [count], that you are going to be okay… and that if you want to be undetectable you have to take all your pills—that's a good message to improve adherence.

It’s the way they express [the message] no? They express it always motivating the youth, the adolescents. And they feel motivated. The [rap] emphasizes a lot taking medications…and that if they take the medication everything will be all right and [the disease] will be controlled.

#### Importance of Content

Adolescents affirmed the importance of the content in the video, particularly messaging that self-care is imperative to a healthy life and that adolescents are responsible for their decisions. Adolescents reported information in the video was important because it encouraged autonomy:

Everything depends on us; taking care of ourselves and improving our health, it all depends on us.

It talks about the medicines, about taking all of the medicines that we have, and it's good because it teaches us not to neglect ourselves, how to treat ourselves, and all of that.

Health providers and adolescents noted the video reinforced that it is possible to live a healthy life with HIV, a message they felt could be especially important during disclosure:

I think it’s perfect. The topic is very interesting because it’s really going to help a lot. I would like to see it … in Ministry of Health materials, something that penetrates, no? Because we would avoid so much. I mean, we would avoid that children [on antiretrovirals] don’t know about these topics…It would help a lot with retention and above all for them to know that there is an incentive …that you are going to be okay, so that is very important. There are many children who get depressed here because of the diagnosis and during the disclosure process they do not accept their diagnosis. (Health provider)

…Because when you receive this news [of the HIV diagnosis], you think that the world suddenly ends, you don't want to live, but with information like this, our emotional state changes, improves, makes you want to continue living. (Adolescent)

It gives hope. It tells you that you are going to be okay. If you take the pills, you will live better, and if you listen to the doctor. So, these messages are positive and so they lift the spirits of the person living with HIV. (Health provider)

#### Planned Uptake and Use

Adolescents reported diverse potential uses of the video. Several mentioned they would watch it to improve their HIV knowledge or lift their spirits. One adolescent explained he would watch the video *“when we feel bad or down… so that it gives me strength*”, while another mentioned it would have been useful to see the video prior to disclosure: “*for [the sake of] our emotional state, no? So that when they give us our diagnosis it doesn’t shock us as much*.” Other adolescents suggested the video could be used in schools for health education or a student-led presentation, as a way to initiate discussion.

Adolescents expressed mixed opinions about sharing the video with friends and family. Proponents of sharing felt it would help their network understand what it was like to live with HIV. Others felt like the sentiment and message of the video would only be understood and appreciated by those living with HIV.

Providers suggested the video could be used to teach ALHIV that it is possible to live a healthy life if adherence to cART is maintained. For example, one pediatrician suggested: *“we should give them the video so they see that there are young people talking about this topic, who are saying that with treatment they can be okay.”* While both providers and adolescents recommended the video be shown in health facility waiting rooms, some providers emphasized the video should be accompanied by dialogue: “*… it is not the same for them to see the video alone as it would be to see it here [in the hospital]. …Perhaps in the hospital with someone from the psychology team or during clinical consultations…”*

#### Themes and Suggestions for Future Videos

When asked about potential themes for future videos, adolescents expressed interest in self-esteem, mental health and emotional wellbeing, basic concepts in HIV/AIDS, HIV prevention, self-care, advances in HIV science, and HIV-related discrimination. Sexual health was also raised as a topic by providers and adolescents. One of the most commonly suggested changes was to vary the music genre. Some participants suggested the repetition of reggaetón music would lend itself to learning lyrics, which for some adolescents were too fast in the current video. Others expressed a preference for slower ballads or bachata music.

### HIV Knowledge

In both pre- and post-video periods, the majority of participants correctly endorsed that cART improved the body’s immune system by increasing CD4 count (24/27, 89%) and missing doses of cART increased HIV in the body (22/27, 81%) (Table 2). The largest improvements in pre- and post-video responses were observed on concepts related to viral load: 7/15 (47%) participants who answered incorrectly in the pre-video questionnaire correctly identified the goal of cART is to decrease viral load and 5/16 (31%) participants identified viral load increases indicate more HIV in the body. Despite the majority of participants answering free-text questions incorrectly, 6/21 (29%) participants who incorrectly defined viral load in the pre-video questionnaire provided a correct definition after watching the video.

**Table 2:** Pre-video and post-video questionnaire responses on HIV literacy (N = 27)

Question	Both incorrect	Pre-correct, post-incorrect	Pre-incorrect, post-correct	Both correct
Is the goal of treatment to make the viral load go up or down?	8 (30%)	2 (7%)	7 (26%)	10 (37%)
If your viral load increases, the amount of HIV in your body has decreased	11 (41%)	0	5 (19%)	11 (41%)
What is a viral load? (free text)	15 (56%)	2 (7%)	6 (22%)	4 (15%)
What is a CD4 count? (free text)	16 (59%)	1 (4%)	5 (19%)	5 (19%)
Your body's ____ fights infections to keep you healthy	14 (52%)	2 (7%)	5 (19%)	6 (22%)
Missing a few doses can increase the amount of HIV in the body	1 (4%)	1 (4%)	3 (11%)	22 (81%)
What is the name of one type of cell that HIV attacks? (free text)	24 (89%)	0	2 (7%)	1 (4%)
When HIV meds work well, the HIV viral load increases	7 (26%)	4 (15%)	5 (19%)	11 (41%)
Is the goal of treatment to make the CD4 count go up or down?	6 (22%)	5 (19%)	5 (19%)	11 (41%)
If HIV meds aren't taken at the right time of day, drug resistance can occur	2 (7%)	3 (11%)	3 (11%)	19 (70%)
What is the name of the type of med a person takes to fight HIV? (free text)	7 (26%)	2 (7%)	1 (4%)	17 (63%)
Your body is best at fighting off infections when your CD4 count is low	3 (11%)	5 (19%)	4 (15%)	15 (56%)
HIV meds help the body's immune system get stronger by increasing CD4	0	3 (11%)	0	24 (89%)

## Discussion

Through questionnaires, focus groups, and in-depth interviews, we found an HIV health literacy music video intervention was widely accepted by ALHIV and their healthcare providers. Qualitative data revealed adolescents felt the video was relatable and familiar due to the selection of actors and filming locations. Adolescents described the video as empowering and provided them with hope they could lead healthy lives. Additionally, some adolescents who were unable to positively identify the role of viral load before the video were able to do so in the post-video questionnaire. These findings demonstrate the need to investigate the impact of delivering health education through music videos on outcomes at the individual and population levels.

We used a rap music video as a medium to deliver HIV-related information to ALHIV. The use of a music video—as opposed to conventional communication like brochures or verbal instruction from providers—fulfills criteria for being an “adolescent-friendly” intervention according to four factors defined by the World Health Organization [28]. First, adolescent-friendly interventions must be *acceptable*, one of the primary aims of our study. Participants described their willingness to re-watch the video to absorb the content or make themselves feel better about their diagnosis, demonstrating acceptability. The World Health Organization emphasizes interventions should also be *equitably* available and *appropriate* for adolescents’ needs [28]. Online video-sharing platforms like YouTube and TikTok are promising distribution mechanisms for video-based health education material considering 94% of youth ages 17–24 in Lima use the Internet [29], primarily on personal cell phones with Internet connectivity [30]. These usage patterns reinforce that educational materials—especially on stigmatizing conditions, like HIV—are ideal for distribution via online videos that can be accessed equitably and viewed privately [29]. Lastly, adolescent-friendly interventions should be *effective* at making positive contributions to adolescent health. Our findings indicate the intervention was both acceptable and feasible, two key components used to determine whether future effectiveness studies should be pursued.

During focus groups, adolescents advocated for the use of the video as a teaching tool in schools. While messages in the study video primarily were targeted at ALHIV, future videos on topics such as HIV prevention and sexual health may be an opportunity to disseminate important health messages to adolescents without HIV. Resources for sexual health information may be particularly valuable in Peru and other settings where a compulsory sexual health education in primary and secondary schools is either not available or not enforced [31]. With regard to other uses of the video, study participants recommended it be played in patient waiting rooms before ALHIV meet with healthcare providers, which could provide an opening for conversation and education. Other adolescents described how the video inspired hope and made them feel they were not alone in their diagnosis, which ALHIV might need especially around the time of diagnosis or disclosure. It may be beneficial to include motivational messaging in future videos on other topics. While a link between health literacy and cART adherence has been established among HIV-infected adults [5–8], the few studies conducted in ALHIV have not identified a significant relationship [32–34]. Understanding the optimal uses of the video intervention will require further studies on the best timing and setting to show the video and potential for pre- and post-video interaction with healthcare providers to reinforce key messages.

A potential limitation in our study is that adolescents received care at the same children’s hospital and were largely perinatally-infected. Because the hospital is the largest pediatric facility in metropolitan Lima, we expect our results are broadly generalizable to other urban Latin American settings and likely extend to all ALHIV, regardless of mode of transmission.

## Conclusion

There is a substantial dearth of evidence on interventions tailored to ALHIV from diverse settings, especially related to improving health literacy. Adolescence is a crucial period to intervene on health literacy as ALHIV gain autonomy and eventually transition from pediatric to adult HIV care, a period marked by poor clinical outcomes and loss to follow up [35]. We found a rap music video focused on HIV-related knowledge was an acceptable and feasible tool to promote health literacy in ALHIV in Lima, Peru. Further investigation is needed on optimal use of this type of tool and whether increases in health literacy translate to improvements in cART adherence and clinical outcomes.
